# In-house environmental factors and childhood acute respiratory infections in under-five children: a hospital-based matched case-control study in Bangladesh

**DOI:** 10.1186/s12887-024-04525-4

**Published:** 2024-01-13

**Authors:** Moktarul Islam, Kariul Islam, Koustuv Dalal, Mohammad Delwer Hossain Hawlader

**Affiliations:** 1 Department of Public Health, North South University, Dhaka, 1229 Bangladesh; 2 School of Health Sciences, Division of Public Health Science, Mid Sweden University, Sundsvall, 851 70 Sweden

**Keywords:** Acute respiratory infections, Under–five children, Environmental factors, Case-control study, Bangladesh

## Abstract

**Background:**

Acute respiratory infection (ARI) is one of the leading causes of morbidity and mortality among children under five globally, particularly in regions like South Asia and sub-Saharan Africa. Bangladesh has made substantial progress in reducing child mortality, yet pneumonia remains a significant contributor to under-five deaths. This study aimed to investigate the association between in-house environmental factors and childhood ARI, considering factors such as household crowding, smoking, and sanitation facilities.

**Methods:**

This case-control study was conducted at a tertiary-level children’s hospital in Dhaka, Bangladesh, from March to September 2019. The study included children aged 6–59 months. Cases were children with ARI symptoms, while controls were children without such symptoms. Rigorous matching by age and gender was employed to ensure comparability. Data were collected through structured questionnaires, and bivariate and conditional logistic regression analyses were performed.

**Results:**

Several household environmental factors were significantly associated with childhood ARIs. Children from overcrowded households (AOR = 2.66, 95% CI = 1.52–4.71; *p* < 0.001), those using unclean cooking fuels (OR = 2.41, 95% CI: 1.56, 3.73; p = < 0.001), those exposed to in-house smoking (AOR = 1.74, 95% CI = 1.01, 3.05; *p* = 0.04) and those with unimproved sanitation facilities faced higher odds (AOR = 4.35, 95% CI = 2.14–9.26) of ARIs. Additionally, preterm birth and higher birth order were associated with an increased risk of ARI. In contrast, exclusive breastfeeding was a protective factor.

**Conclusion:**

In-house environmental factors, including sanitation, crowding and in-house smoking, significantly influence childhood ARIs. Additionally, birth order and preterm birth play a crucial role. Promoting exclusive breastfeeding is associated with a lower ARI risk among under-five children in Bangladesh. These findings can guide interventions to reduce ARIs in low-income regions, particularly in South Asia.

## Introduction

Acute respiratory infections (ARIs) continue to be a leading cause of morbidity and mortality among children under the age of five worldwide [1]. The World Health Organization (WHO) estimates that, in 2022, there were 5 million deaths among children under the age of five caused by ARIs, which could have been prevented or treated. ARI is now a global problem of health policy interests [1, 2], accounting for approximately 20% of children’s deaths globally, with a significant proportion occurring in South Asia and sub-Saharan Africa. Acute respiratory infections (ARIs) account for 3.5% of the worldwide disease burden. They were the cause of 30–50% of all pediatric outpatient visits and resulted in over 30% of pediatric admissions in low- and middle-income countries [1]. According to UNICEF, in 2019, pneumonia alone was responsible for approximately 50% of under-five children’s deaths from all infectious diseases, indicating above 700,000 death tolls globally [2] and 20% of all deaths worldwide, mostly in South Asia and sub-Saharan Africa [3, 4]. Multiple studies have shown that Bangladesh, India, Indonesia, and Nepal are responsible for 40% of children’s deaths due to ARIs [5, 6]. The case fatality of severe pneumonia was found to be increasing while concomitant with measles infection and contributing to approximately 38% of measles deaths [7].

Bangladesh successfully achieved the Millennium Development Goal (MDG) 4 by significantly reducing the child mortality rate to 46/1000 live births by 2015 and it reduced to 45/1000 live births in 2017-18 [8]. According to Bangladesh Sample Vital Statistics 2018, the major cause of death is pneumonia in children under the age of five, accounting for more than one-third of all deaths (34.4%) [9]. To achieve the target set by the Integrated Global Action Plan for the Prevention and Control of Pneumonia and Diarrhea (GAPPD) by 2025, which aims to reduce mortality from pneumonia in under-five children to fewer than 3 per 1000 live births [3].

Several factors increase the risk of contracting and succumbing to acute respiratory infection (ARIs). These factors included poverty, malnutrition, low birth weight, inadequate breastfeeding, complementary food initiation, overcrowding, poor living conditions, insufficient sanitation, exposure to indoor and outdoor pollution, seasonal changes, and limited access to preventive and curative services [5, 6, 10, 11]. Recent observational studies found that household environmental factors, such as smoking, poor water quality, high dwelling density, and a lack of toilet facilities, contributed significantly to childhood acute respiratory infections (ARIs) [12, 13].

Multiple studies explored the role of different determinants of early childhood ARI in Bangladesh [14–17]. However, most of these studies had limitations due to their cross-sectional designs. Another issue was the need for more data on all potential predictors representing the diverse dimensions. Even though the investigations of multiple studies on whether environmental factors were potential risk factors for ARI [3, 5, 6, 10, 11], there was no clear evidence regarding the association with in-house environmental factors, especially for in-house toilet facilities, indoor smoking and crowding on ARI among children under-five years of age in our country. Hence, the study’s primary aim was to observe the association between in-house environmental factors where the children were nested and ARI. The study’s findings will give us the understanding to focus on the required areas to stem the problem in low-income settings of South Asia by advocating the improvement of behavioral and programmatic intervention programs.

## Methods

### Study design and sampling technique

We conducted a hospital-based matched case-control study at a specialized government-run tertiary children’s hospital (Dhaka Shishu Hospital) in Dhaka, Bangladesh, between March and September 2019. The study population consisted of children aged 6–59 months. We employed a rigorous matching process to ensure the comparability of cases and controls. Specifically, for each case (children with ARI), we selected a control from the same hospital, individually matched by age and gender. This matching process involved identifying a control who belonged to the same age category and shared the same gender as the corresponding case. In essence, each case was precisely paired with a control of the same age and gender, thus minimizing potential confounding variables. This stringent matching strategy was crucial to ensuring the comparability of cases and controls throughout the study. Study population.

Cases were defined as children aged 6–59 months who were hospitalized in the pediatric department with specific symptoms, including mild running nose, cough, chest in-drawing, and fast and difficult breathing (11) and diagnosed by the physicians of the hospital using the ‘Integrated Management of Childhood Illness protocol’ [18]. In contrast, controls consisted of the children aged 6–59 months who had attended the outpatient department without ARI symptoms, and the healthy children accompanying the patients to the same hospital and having no ARI symptoms in the previous 30 days were defined as the control population. The children with compromised immune systems diagnosed in the past were not involved in the study to minimize any potential effect on the study results.

### Sample size estimation

The sample size of the study was calculated using the following formula, assuming a 5% significance level (i.e., $${Z}_{\raisebox{1ex}{$\alpha $}\!\left/ \!\raisebox{-1ex}{$2$}\right.}$$=1.96), 80% power (i.e., $${Z}_{\beta }$$ = 0.84) of the study, and a case-control ratio of 1:1 (*r*=1). The percentage of control under five years of age exposed to overcrowding was assumed to be 30.5% (i.e., $${p}_{2}$$=0.305) with an odds ratio (OR) of 2.06 based on an analytical cross-sectional study done in India [19].

$$n=\left(\frac{r+1}{r}\right)\frac{\stackrel{-}{p}\left(1-\stackrel{-}{p}\right){\left({Z}_{\beta }+{Z}_{\raisebox{1ex}{$\alpha $}\!\left/ \!\raisebox{-1ex}{$2$}\right.}\right)}^{2}}{{\left({p}_{1}-{p}_{2}\right)}^{2}}$$

Therefore, based on the standard statistical parameters indicated above, our study was initially planned with a minimum sample size of 141 cases and an equal number of controls. However, the availability of facilities and resources allowed us to expand our sample size to 348, comprising 174 cases and 174 controls. This increase in the sample size was a strategic decision taken to enhance the statistical power of our study. A larger sample size improves the reliability of our results and increases our sensitivity to identifying relationships.

### Data collection technique and quality control

Once the parents or other caregivers of the children had provided written informed consent, the skilled and widely trained data collectors administered a semi-structured questionnaire (described in both English and Bengali) for data collection through frontal interviews. For both the case and control groups, we utilized a single questionnaire utilizing national standard tools [20, 21] and maintained WHO-recommended procedures, [22] and the data collected were checked and rechecked for their reliability and validity.

### Outcome variable

Acute respiratory infection (ARI) was defined as the specific symptoms that were onset in the preceding 10 days from the day of the visit, including a mild running nose, cough, chest in-drawing, and fast and difficult breathing.

### Independent variables

Data were collected on three sets of predictors: household environmental (exposure variables), sociodemographic, and mother-child characteristics.

### Household environmental (exposure variables)

A group of variables was set for analysis to determine the effect of the household environment on ARIs among children under five. It included the place of residence (urban, rural), in-house crowding, in-house smoking habits by family members, cooking fuel, source of drinking water, shared toilets, and toilet facilities. The child’s residence had been identified as whether he/she was part of urban or rural living. In-house crowding was described as the total number of household members divided by the total number of bedrooms [23]. The number of members of the household was counted, asking, “How many members stay with the child in your house?” We categorized the in-house crowding into two following groups: >3 and ≤ 3 people per bedroom [13]. We assumed the cut point of 3.0 for in-house crowding categorization because we thought the child was staying with his/her parents.

We further investigated several household environmental variables: the type of cooking fuel used and the in-house smoking habits of the family members in the house. The use of cooking fuel was classified into two groups: clean (natural gas, LPG or electricity) or unclean (biogas, wood, agricultural products, solid waste or coal) [24]. In-house smoking was determined by asking, “Do any of the adult members of your family smoke on the home premises?” We also took data on their source of drinking water, which was recorded as either tap or piped water or tube-well or surface water. In addition, two kinds of data were obtained from the provision of sanitation facilities in the house in which the children stayed. First, whether or not they used a communal toilet (more than five people use the same toilet) and second, the type of toilet they were using. The toilets were divided into two categories: improved (safe disposal of excreta without human contact, e.g. flush to the piped sewage system, septic tanks or pit latrines) and unimproved facilities (pit latrine without slab/platform, hanging/bucket toilet or open) [25].

### Other covariates

The sociodemographic variables consisted of the child’s age (in months) and gender, the level of education for both parents, the mother’s occupation, and the family’s monthly income. Parental education was categorized by years of schooling (illiterate, 1–5, 6–12, and 12 + years). The mother’s occupation was classified as either a housewife or employed. We defined the employed mothers as those who received a monthly salary from their occupation. Family income was classified into three groups ( < = 10000, 10,001–25000, > 25000 BDT/month).

Last, the maternal-child characteristics included birth order, gestational age (preterm/full-term), mode of delivery (cesarean section/normal), exclusive breastfeeding, nutritional status, and vaccination status (based on EPI schedule; completeness for age or no/incomplete). Preterm birth is defined as the birth of a child before completing 37 weeks of gestation, and full-term birth refers to a birth after 37 weeks [26]. Nutritional status was assessed by the mid-upper arm circumference (MUAC) and categorized into two groups: healthy (> 13.5 cm) and malnourished ( < = 13.5 cm). It was measured by MUAC (mid-upper arm circumference) tape at the midpoint between the tip of the shoulder and the tip of the elbow. The measurements were meticulously recorded with a precision of 0.1 cm. The tape fitted firmly but did not create any pit in the upper arm.

### Statistical analysis

The questionnaires analyzed were checked for completeness, precision, and internal consistency, and the exclusion of incomplete or inaccurate data. The analytical software STATA was used to analyze the data. The descriptive statistics were presented as percentages and frequencies. We conducted bivariate and multivariate conditional logistic regression analyses to examine the association between in-house environmental factors and childhood ARI. The study employed a matched case-control design, where each case (children with ARI) was matched with a control (children without ARI) based on age and gender. This matching was essential to control for potential confounding variables and to ensure that cases and controls were comparable. Conditional logistic regression was well-suited for analyzing matched data because it takes into account the matched pairs and their characteristics. Furthermore, the outcome variable in this study was binary (ARI present or not present), which was suitable for logistic regression analysis. Conditional logistic regression further extended logistic regression to matched data, allowing researchers to examine the relationship between the predictors (in-house environmental factors, sociodemographic variables, etc.) and the binary outcome while accounting for the matching.

We built our regression models iteratively, considering variables’ significance and potential confounding effects. Collinearity among all independent variables included in the study was assessed using the Variance Inflation Factor (VIF). Apart from “*residence*”, no other variable exhibited significant collinearity. As a result, these were retained and included in the subsequent conditional logistic regression model. This allowed us to accurately examine the relationship between these variables and the binary outcome. In both models, odds ratios (ORs) with 95% confidence intervals (CIs) were used to assess the strength and direction of associations and a *p*-value < 0.05 was considered to determine statistical significance.

### Result

The analysis considered three hundred and forty-eight children aged 6 to 59 months, with 174 cases and 174 controls. As a matched case-control study by age and gender, the distribution of boys (60.34%) and girls (39.66%) was similar in both the case and control groups. Most participants in each group were between the ages of 25–36 months (39.66% of cases and 39.08% of controls).

### Association of sociodemographic characteristics with childhood ARI

We examined the impact of various sociodemographic characteristics on childhood ARI (acute respiratory infections) in the bivariate logistic regression model [Table 1]. Approximately half of the mothers (47.13% for cases and 50.57% for controls) completed 6–12 years of schooling. The correlation between maternal education and childhood ARI was almost significant (*p* = 0.051), with children of mothers who completed 6–12 years of schooling having 39% lower odds of ARI compared to those with less-educated mothers. Almost 40% of the fathers and 23% of mothers completed more than 12 years of schooling. However, a closer examination revealed that children of more highly educated mothers (12 + educational years) and fathers with secondary education (6–12 years of schooling) demonstrated substantial decreased odds of childhood ARI by 66% (OR = 0.44, 95% CI: 0.24, 0.81; *p* = 0.008) and 76% (OR = 0.34, 95% CI: 0.15, 0.72; *p* = 0.006) respectively, relative to illiterate parents (reference category).

Furthermore, Approximately 52% of children were nested in households with monthly family income between BDT 10,001 and BDT 25000 in both cases (50.57%) and control (52.87%)groups. Children from families with a monthly income exceeding 25,000 BDT per month displayed 50% lower odds (OR = 0.50, 95% CI: 0.26, 0.94; *p* = 0.033) of ARIs than those from families with monthly incomes 10,000 BDT or less. However, the mother’s occupation did not exhibit any significant association with childhood ARI [Table 1].

**Table 1:** Association of sociodemographic characteristics with childhood ARI: Unadjusted logistic regression model

Characteristics	Case *N* = 174(%)	Control *N* = 174 (%)	Unadjusted OR (95% CI)	*p* value
**Child gender**				
Male	105 (60.34)	105 (60.34)	Matched variable	
Female	69 (39.66)	69 (39.66)		
**Child age (months)**				
6–12	22 (12.64)	17 (9.77)	Matched variable	
13–24	69 (39.66)	68 (39.08)		
25–36	39 (22.41)	39 (22.41)		
37–48	23 (13.21)	29 (13.22)		
49–59	21 (12.07)	21 (12.07)		
**Mother’s education**				
No Schooling/1–5 years	60 (34.48)	39 (22.41)	Reference	
6–12 years of schooling	82 (47.13)	88 (50.57)	0.61 (0.37, 0.99)	0.051
12 + years of schooling	32 (18.39)	47 (27.01)	0.44 (0.24, 0.81)	0.008*
**Father’s education**				
Illiterate	25 (14.37)	14 (8.05)	Reference	
1–5 years of schooling	43 (24.71)	26 (14.94)	0.93 (0.40, 2.08)	0.854
6–12 years of schooling	38 (21.84)	63 (36.21)	0.34 (0.15, 0.72)	0.006*
12 + years of schooling	68 (39.08)	71 (40.80)	0.54 (0.25, 1.10)	0.096
**Mother’s occupation**				
Employed	13 (7.47)	11 (6.32)	Reference	
Housewife	161 (92.53)	163 (93.68)	0.84 (0.36, 1.92)	0.673
**Family monthly income**				
**≤** 10000	60 (34.48)	44 (25.29)	Reference	
10,001–25000	88 (50.57)	92 (52.87)	0.70 (0.43, 1.14)	0.153
> 25,000	26 (14.94)	38 (21.84)	0.50 (0.26, 0.94)	0.033*

*significant at a 5% significance level

OR: odds ratio; CI: confidence interval

### Association of in-house environmental factors with childhood ARI

We also investigated the influence of household environmental factors on ARIs among children aged 6–59 months, as demonstrated in the unadjusted model [Table 2]. The residence of the children exhibited a significant association with childhood ARI. Specifically, children residing in rural areas were more than twice as likely (OR = 2.12, 95% CI: 1.38, 3.28; *p* = 0.001) to experience ARIs compared to their urban counterparts (reference category). In particular, our data revealed that 53.45% of households in the case group had more than three individuals per bedroom, which was significantly higher than the 24.13% observed in the control group. Children living in overcrowded households with more than three people per bedroom faced 3.61 times higher odds of developing acute respiratory infections (ARIs) when compared to those in less crowded households with three people or fewer per bedroom (OR = 3.61, 95% CI: 2.29, 5.74; p = < 0.001) in the unadjusted model. Furthermore, the use of unclean fuel by families increased the odds of ARIs among children by 2.41 times (OR = 2.41, 95% CI: 1.56, 3.73; p = < 0.001) compared to those using clean fuels. Additionally, a significant proportion of family members in both case and control categories reported in-house smoking habits (40.23% in cases and 29.89% in controls). In households with in-house smokers, the risk of childhood ARI significantly increased by 58% when compared to households with nonsmokers or those without in-house smoking (OR = 1.58, 95% CI: 1.02, 2.47; *p* = 0.04). Approximately 45% of families relied on tube-well or surface water for drinking, which was associated with 2.45 times higher odds of childhood ARI (OR = 2.45, 95% CI: 1.59, 3.79; p = < 0.001) compared to those consuming tap or piped water. Encouragingly, approximately three-fourths (75.86%) of families used improved sanitation facilities, signifying a positive trend. However, the utilization of unimproved toilet facilities significantly increased the risk of childhood ARI by 5.29 times (OR = 5.29, 95% CI = 3.03, 9.66; p = < 0.001). Interestingly, the sharing of toilet facilities did not yield a significant increase in the risk of childhood ARI [Table 2].

**Table 2:** Association of house-environmental factors with childhood ARI: Unadjusted logistic regression model

Characteristics	Case n = 174 (%)	Control n = 174 (%)	Unadjusted OR (95% CI)	*p*-value
**Residence**				
Urban	62 (35.63)	94 (54.02)	Reference	
Rural	112 (64.37)	80 (45.98)	2.12 (1.38, 3.28)	0.001*
**In-house crowding**				
**≤** 3	81 (46.55)	132 (75.86)	Reference	
> 3	93 (53.45)	42 (24.13)	3.61 (2.29, 5.74)	< 0.001*
**Cooking fuel**				
Clean	79 (45.40)	116 (66.67)	Reference	
Unclean	95 (54.59)	58 (33.33)	2.41 (1.56, 3.73)	< 0.001*
**In-house Smoking habit by family members**				
No	104 (59.77)	122 (70.11)	Reference	
Yes	70 (40.23)	52 (29.89)	1.58 (1.02, 2.47)	0.04*
**Source of drinking water**				
Tap/piped water	76 (43.68)	114 (65.52)	Reference	
Tube well/surface water	98 (56.32)	60 (34.48)	2.45 (1.59, 3.79)	< 0.001*
**Toilet facilities**				
Improved	108 (62.07)	156 (89.66)	Reference	
Unimproved	66 (37.93)	18 (10.34)	5.29 (3.03, 9.66)	< 0.001*
**Shared toilet**				
No	120 (68.97)	129 (74.14)	Reference	
Yes	53 (30.46)	45 (25.86)	1.27 (0.79, 2.03)	0.324

*significant at a 5% significance level

OR: odds ratio; CI: confidence interval

### Association of maternal and child characteristics with childhood ARI

We then assessed the effect of various maternal and child characteristics on childhood ARI in the bivariate logistic regression model [Table 3]. The data indicated that most children were firstborn in the family (33.91% among cases and 48.28% among controls). An intriguing trend became apparent – the odds of ARI increased with ascending birth order. The first- and second-born children had an effective defence against ARI related to third- or more-ordered children (OR = 0.31, 95% CI: 0.19, 0.58; p = < 0.001 and OR = 0.38, 95% CI: 0.22, 0.69; *p* = 0.002, respectively). Most children were born after 37 weeks of gestation (54% in the case group and 68.39% in the control group). Remarkably, premature birth amplified the odds of ARI by a substantial 84% (OR = 1.84, 95% CI: 1.19, 2.86; *p* = 0.006) compared to full-term (reference category). Only 56.32% of all children received exclusive breastfeeding. However, children who breastfed exclusively were 50% (OR = 0.50, 95% CI: 0.32, 0.77; *p* = 0.002) less likely to develop ARI than those not. Most children completed vaccination for age (79.31% for the group of cases and 92.53% for the group of controls). The odds ratio indicated that children who did not receive immunization by the recommended age were 3.5 times (OR = 3.50, 95% CI: 1.80, 7.26; *p* < 0.001) more inclined to develop ARI in comparison to their counterparts who received completely (the reference category). The majority of all children (47.13%) were delivered through the typical vaginal route (52.30%). In a bivariate model, the nutritional status and mode of delivery among under-five children were not significantly correlated with ARI [Table 3].

**Table 3:** Association of children and maternal characteristics with childhood ARI: Unadjusted logistic regression model

Characteristics	Case *N* = 174 (%)	Control *N* = 174 (%)	Unadjusted OR (95% CI)	*p*-value
**Birth order**				
≥ 3 born	61 (35.06)	27 (15.52)	Reference	
2nd born	54 (31.03)	63 (36.21)	0.38 (0.22, 0.69)	**0.00** 2*
1st born	59 (33.91)	84 (48.28)	0.31 (0.19, 0.58)	< 0.001*
**Gestational age**				
Full term	94 (54.02)	119 (68.39)	Reference	
Preterm	80 (45.98)	55 (31.61)	1.84 (1.19, 2.86)	0.006*
**Mode of delivery**				
C-section	83 (47.70)	101 (58.05)	Reference	
Normal	91 (52.30)	73 (41.95)	1.52 (0.99, 2.32)	0.054
**Exclusive breastfeeding**				
No	86 (49.43)	58 (33.33)	Reference	
Yes	83 (47.70)	113 (64.94)	0.50 (0.32, 0.77)	**0.00** 2*
**Nutritional Status**				
Malnourished	54 (31.03)	34 (19.54)	Reference	
Healthy	80 (45.98)	59 (33.91)	0.85 (0.49, 1.47)	0.570
**Vaccination**				
Complete for age	138 (79.31)	161 (92.53)	Reference	
No/Incomplete for age	36 (20.69)	12 (6.90)	3.50 (1.80, 7.26)	< 0.001*

*significant at a 5% significance level, C-section: cesarean section

OR: odds ratio; CI: confidence interval

### Conditional logistic regression: an adjusted model

We finally conducted a multivariable conditional logistic regression analysis to estimate the adjusted odds ratios (AORs) for identifying potential in-house environmental factors. After adjusting the effect of household, maternal and child characteristics, we found that several factors remained significantly associated with childhood ARIs [Table 4].

Unimproved toilets were significantly associated with increased odds of childhood ARI by 4.35 times (AOR = 4.35, 95% CI = 2.14, 9.26; *p* < 0.001)). Crowded households with more than three people per bedroom also showed a significantly elevated risk of childhood ARI by 2.66 times while all other variables were kept constant (AOR = 2.66, 95% CI = 1.52, 4.71; *p* < 0.001) while adjusting the other covariates. Likewise, households with family members who smoked indoors also demonstrated increased odds of childhood ARIs by 1.74 times (AOR = 1.74, 95% CI: 1.01, 3.05, *p* = 0.04).

The adjusted odds of childhood ARI were also found to be significant among maternal and child characteristics in the conditional logistic regression model; in terms of preterm birth by 2.44 times (AOR = 2.44, 95% CI: 1.43, 4.23; *p* < 0.001) (AOR = 2.44, 95% CI: 1.43, 4.23; *p* < 0.001) and having three- or more-birth ordered children by 4.26 times (AOR = 4.26, 95% CI = 2.07, 9.03; *p* < 0.001), and in-house smoking (AOR = 1.74, 95% CI = 1.01, 3.05; *p* = 0.04).

In addition, exclusive breastfeeding alone significantly minimizes ARI in under-five children (AOR = 0.48, 95% CI = 0.28, 0.82; *p* = 0.01) [Table 4].

**Table 4:** Association of potential in-house environmental and maternal factors with childhood ARI: Adjusted Conditional logistic regression model

Characteristics	Reference	Adjusted OR (95% CI)	*p*-value
**Mother’s education**			
6–12 years of schooling	No/1–5 years schooling	0.83 (0.43, 1.63)	0.60
12 + years of schooling		1.55 (0.59, 4.17)	0.38
**In-house crowding**			
> 3	<=3	2.66 (1.52, 4.71)	< 0.001*
**Source of water**			
Tube well/surface water	Tap/piped water	1.27 (0.64, 2.51)	0.50
**Cooking fuel**			
Unclean	Clean	1.77 (0.9, 3.47)	0.10
**Toilet facilities**			
Unimproved	Improved	4.35 (2.14, 9.26)	< 0.001*
**In-house Smoking habit by family members**			
Yes	No	1.74 (1.01, 3.05)	0.04*
**Birth order**			
≥ 3 born	1st born	4.26 (2.07, 9.03)	< 0.001*
2nd born		1.15 (0.63, 2.09)	0.64
**Gestational age**			
Preterm	Full term	2.44 (1.43, 4.23)	< 0.001*
**Exclusive breastfeeding**			
Yes	No	0.48 (0.28, 0.82)	0.01*
**Vaccination**			
No/Incomplete for age	Complete for age	1.76 (0.77, 4.18)	0.19

*significant at a 5% significance level

AOR: adjusted odds ratio; CI: confidence interval

## Discussion

This case-control study, individually matched by age and gender- was conducted at a tertiary children’s hospital in Bangladesh and aimed to investigate the association of in-house environmental factors and acute respiratory infection among children aged 6–59 months in the country’s population where we identified that several household environmental factors had a critical role to developing childhood acute respiratory infection (ARI). The study encompassed 348 participants and performed both bivariate and multivariate conditional logistic regression models to explore the associations.

Our study observed a substantial increase in ARI among children living in - crowded households with more than three persons per bedroom. Specifically, our findings indicated a 2.12-fold increase in the odds of ARI in such conditions (OR = 2.12, 95% CI: 1.38, 3.28). Even after adjusting all other variables, it remained unchanged. This aligned with previous research in Canada, which demonstrated a similar association where indoor CO_2_ where indoor CO2 levels increased with the number of indoor occupants, significantly elevating the risk of respiratory infections among young children [27]. Congested living conditions made spreading infections easier, increasing the risk of cross-infection when family members sneezed, coughed, or talked. Such houses were mostly ill-ventilated [28]. Studies revealed that poor ventilation in the house increases the odds of ARIs in young children [29, 30]. However, indoor humidity was also influenced by reduced out-to-indoor airflow rates, which augmented the growth of microorganisms in the house, increasing the risk of ARI in the susceptible group [31]. The result of the study was consistent with other countries, such as India and Nigeria, where the variable overcrowding in the house was considered [19, 32].

The present study also shed light on a significant association between unimproved toilet facilities and the increased odds of childhood ARI by 4.35 times (AOR = 4.35, 95% CI: 2.14, 9.26) after adjusting for the effect of other covariates. Our findings aligned with studies conducted in similar settings, including Bangladesh, India and Pakistan, which consistently reported similar results [17, 19, 33]. Unimproved toilets were primarily prevalent in rural areas where access to modern sanitation facilities was often limited or unavailable. These unimproved facilities were typically utilized by people with lower levels of education and financial means [34, 35]. The lack of access to improved sanitation not only increases the risk of ARIs but also contributes to a broader public health challenge by facilitating the transmission of infectious diseases.

In Bangladesh, young children generally accompany their mothers while cooking. In our study, dirty cooking fuel was found to be significantly associated with ARIs. Such fuels were mostly used in rural areas. The analysis revealed higher odds of ARIs among children of rural dwellings than among children of urban dwellings and the findings were compatible with several studies [19, 36]. Studies have also shown that irritant air pollutants, such as particulate matter, carbon monoxide, nitrogen dioxide, sulfur dioxide, and formaldehyde, are produced more in solid biomass fuel combustion than in clean fossil fuels. These toxic air pollutants generated within the house become trapped and have the potential to increase microbial infection susceptibility [37]. A meta-analysis conducted with the studies in rural households showed that exposure to solid biomass fuel significantly increased the risk of ARI among children by 3.53 times [38].

The current study also brought to light the adverse impact of exposure to second hand smoke in children, increasing their susceptibility to acute respiratory infections (ARIs). This association was consistent with findings from various regions, Nepal, Bangladesh, and Cameroon [10, 33, 36]. Second hand smoke, often found in households where family members smoke indoors, can impair the natural protective mechanisms against ARI in children [39]. However, it is noteworthy that not all studies reported such a link. A notable exception was found in the study conducted in Ethiopia [37], which did not find a significant association. Contextual factors, such as smoking prevalence and cultural practices, may influence these variations in findings.

Furthermore, our findings aligned with a recent meta-analysis that revealed a concerning association. Pregnant women exposed to passive smoking at home amplify the risk of delivering preterm babies [40]. Preterm children were particularly vulnerable to infections [41]. The present study observed a 2.44-field elevated odds of ARI among the children with preterm birth (AOR = 2.44, 95% CI: 1.43, 4.23. Additionally, our findings underlined the heightened susceptibility of premature babies to infections, particularly during their first year of life [42]. Preterm birth, a complex and multifaceted issue, is associated with an increased risk of various health challenges, including a higher likelihood of developing acute respiratory infections (ARIs). Our study indeed aligned with these trends as we observed significantly higher odds of ARI among preterm children under the age of five in a study conducted in India [11].

Our study findings emphasized the vital role of parental education in reducing the occurrence of childhood ARIs. The health and well-being of children are intricately linked to their caregivers’ knowledge, attitudes, and practices regarding water, sanitation and hygiene (WASH) [43]. Studies from various regions have consistently demonstrated the protective association between parental education and a reduced risk of ARI. For instance, research conducted in Nigeria found a protective correlation between childhood ARI and parental education [32]. Similarly, a study encompassing several developing nations indicated that maternal education played a pivotal role in reducing the risk of childhood ARI [44].

Our study showed that a family with a relatively high income (exceeding 25,000 BDT/month) had significant protection against ARI among children under five in conditional logistic regression analysis. However, in-house environmental factors were regarded as an indication of social and economic disadvantage [45]. Individuals with economic constraints were usually forced to live in a house with insufficient household environmental facilities for their necessities. Previous studies from Bangladesh also revealed such results [14–17].

In our study, we found that those who were exclusively breastfed, properly immunized for age, and had fewer than two siblings (in cases of multiple births) had significantly lower odds of ARI in childhood. Studies from different countries have provided similar results [10, 32, 36]. Having more siblings reduces the mothers’ attention on individual children, thus increasing the chance of different diseases. Immunoglobulins in breast milk help significantly reduce the risk of ARI in children. The healthy children had fewer odds of ARI, but the association was not significant, which contrasts with the findings of other countries [36, 46].

Improving home ventilation is a low-cost strategy. It involves simply opening windows and doors to disperse excess CO_2_ and create an environment less conducive to the growth of harmful microorganisms. Additionally, discouraging indoor smoking is another low-cost measure that can reduce ARIs in children and help mitigate the risk of premature birth, which is also independently associated with ARIs. Supporting this, continuation of breastfeeding for at least six months and offering guidance to mothers with larger families is essential. Furthermore, the population-based approach prioritizes health education, especially in rural areas, to reduce reliance on unclean cooking fuels and promote improved sanitation facilities. Complementing these efforts, broader initiatives aimed at poverty reduction, social equity and establishing a supportive social welfare system can ensure that residents are not compelled to live in overcrowded, inadequate spaces due to sudden financial shocks.

Our analysis had some limitations that need to be acknowledged. It was tertiary hospital-based research and, therefore, did not highlight the hidden aspect of the iceberg epidemic, including mother-child pairs who could not use hospital facilities or even primary health care. Recruiting controls at the hospital may have introduced a self-selection bias. Future research should use a longitudinal sample to confirm the causal relations between public and private hospitals nationwide. Further, research could comprehensively explore the causal relationships between various factors and ARI incidence.

## Conclusion

The study hypotheses were adequately tested to confirm that in-house environmental factors in Bangladesh have an apparent, significant influence on childhood ARI, especially unimproved household sanitation facilities, in-house crowding (children living in a house where more than three people live in a bedroom), and in-house smoking, which were found to be important influencers in developing acute respiratory infection among under-five children. In addition, higher birth order and preterm birth were also found to play an important role in the development of ARI. Conversely, exclusive breastfeeding might significantly reduce ARIs in children under five in Bangladesh.

## Data Availability

The data underlying the results presented in this study will be provided upon reasonable request to Dr Delwer H. Hawlader. Email: mohammad.hawlader@northsouth.edu.

## Abbreviations

ARI: Acute Respiratory Infections
WHO: World Health Organization
UNICEF: United Nations Children’s Fund
MDG: Millennium Development Goal
GAPPD: Global Action Plan for the Prevention and Control of Pneumonia and Diarrhea
OR: Odds Ratio
AOR: Adjusted Odds Ratio
CI: Confidence Interval
MUAC: Mid-Upper Arm Circumference
EPI: Expanded Program on Immunization
VIF: Variance Inflation Factor
LPG: Liquefied Petroleum Gas
BDT: Bangladeshi Taka
C-section: Cesarean Section
